# Teachers’ attitudes to cultural diversity: Results from a qualitative study in Russia and Taiwan

**DOI:** 10.3389/fpsyg.2022.976659

**Published:** 2022-11-16

**Authors:** Jui-Sheng Wang, Jesse Yu-Chen Lan, Rezeda R. Khairutdinova, Chulpan R. Gromova

**Affiliations:** ^1^Department of Accountancy and Graduate Institute of Finance, National Cheng Kung University, Tainan, Taiwan; ^2^Department of Public Policy and Management, I-Shou University, Kaohsiung, Taiwan; ^3^Institute of Psychology and Education, Kazan (Volga Region) Federal University, Kazan, Russia

**Keywords:** teacher attitudes, cultural diversity, immigrant children, multiculturalism, ethnic

## Abstract

This study examined elementary school teachers’ attitudes toward immigrant students and their families in Russia and Taiwan. Qualitative methodology was used for data collection. Teachers’ attitudes and conceptual orientations toward cultural diversity in the classroom were identified. Teachers’ attitudes were categorized into three groups: Attitudes toward children, parents, and diverse ethnic groups. The study found that country-specific attitudes were similar. Both countries prioritize cognition. The Russian teacher may not acknowledge cultural variance (personal attitude) and hold all students to the same standards (professional attitude). Prior experiences have made the Taiwanese instructor more accepting of cultural variety. Teachers of immigrant children often tackle language concerns. Some instructors are less welcoming of immigrants at home. Educators judge immigrants depending on their natal country. These impressions may encourage prejudice, some say. Many teachers dislike minority students. Negative attitudes concerning student achievement lead to low expectations. Instructors use more tolerant approaches with more multicultural engagement.

## Introduction

Given the increasing demographic diversity in many countries, much research has specifically addressed the impact of cultural diversity on schools. School environments foster positive intercultural relationships among students and teachers of diverse backgrounds. Research on multicultural education indicates that teachers’ responses to increasing cultural diversity in schools worldwide are of central importance (Sleeter, 2001; Gay, 2002; Gorski, 2009; Banks and Banks, 2019). For example, Banks (2001) emphasized that teachers must understand, accept, and respect diverse ethnic groups and various culturally specific manifestations in the classroom to meet all students’ educational and cultural needs. Teachers’ attitudes influence their classroom practices and strategies (Gay, 2002; Garmon, 2004; Sinkkonen and Kyttälä, 2014) and affect students’ psychological, social, and academic adjustment (Birman and Tran, 2017). Therefore, teachers’ attitudes toward cultural diversity are critical to the “change needed for teachers to implement appropriate instruction and enact curriculum relevant to all students” (Harrington and Hathaway, 1995).

Russia has always been a multiethnic society and currently receives large numbers of immigrants (United Nations, 2015). According to immigration data, Russia attracts mostly labor migrants, with the largest source countries of immigration being Ukraine (31%), Kazakhstan (12%), Uzbekistan (11%), Tajikistan (9%), Armenia (8%), Kyrgyzstan (5%), Azerbaijan (4%), and China (2%; MIARF, 2019). Family migration presents new challenges in integrating immigrant children into Russian society. Researchers and educators are looking for evidence-based ways to adapt and acculturate immigrant children through the education system.

In Taiwan, according to statistics from the Taiwan Ministry of Labor at the end of 2011, Vietnamese nationals represented the largest number of migrant workers, about 205,000, accounting for 46% of total migrant workers, followed by 115,000 (26%) from the Philippines, and 65,000 from Indonesia (15%) and 56,000 in Thailand (13%). Bhopal (2011) emphasized that teachers’ attitudes toward students from diverse cultural backgrounds depend on the interaction context (school or society). Teachers’ attitudes toward cultural diversity in society can be changed through everyday pedagogical practice. Therefore, this study aims to identify and classify teachers’ attitudes toward cultural diversity in the classroom and children with immigrant backgrounds. The study addresses the following research questions:

1)What are the main types and characteristics of teachers’ attitudes toward cultural diversity?
2)How are teachers’ academic expectations of immigrant children reflected in their attitudes?
3)How is the conceptual orientation of teachers’ attitudes toward cultural diversity expressed in the classroom?

### Theoretical framework

Before we can make sense of cultural diversity, we must first define culture. Earley and Gibson (2002) describe culture as the social setting’s overlapping expectations, responsibilities, and regulations. Culture may also be understood as a group trait that develops over time (Earley and Mosakowski, 2000). In a homogenous and isolated community, a cultural structure can be retained. In today’s world, however, with migration, social contact, and internet technologies all having a significant influence, the likelihood of cultural structures being unique and conserved is quite low.

In many nations, cultural diversity is viewed as ethnic diversity (Holm and Londen, 2010; Richeson and Sommers, 2016), yet it is defined in a broader meaning. Cultural diversity can be defined as diversity in terms of social class, ethnicity, language, race, gender, sexual orientation, ability/disability, and religion (Holm and Londen, 2010; Michael-Luna and Marri, 2011). Fylkesnes (2018) investigated how the phrase “cultural diversity” is utilized in educational research. According to the study’s findings, education scholars assign different levels of significance to various aspects of cultural diversity depending on their positions. Also, it has been connected to teaching founded on racial fairness.

Cultural diversity is associated with multicultural education. It is a democratic method of teaching and learning that aims to encourage cultural diversity among culturally varied cultures and a global community that is more interdependent (Guyton and Wesche, 2005). The concept of multiculturalism refers to the acknowledgment of the unique characteristics of several cultures and the coming together of those cultures into a single entity. The concept of multiculturalism refers to situations in which many aspects of culture, including but not limited to racial and ethnic diversity, sexual orientation, gender, age, social status, educational attainment, and religious or spiritual leanings, are acknowledged (Karacabey et al., 2019).

Teachers need to be aware of how to teach in a way that is culturally sensitive for multiculturalism to be successfully implemented in educational environments (Abacioglu et al., 2020). Culturally responsive teaching, which is defined as making use of the cultural knowledge, prior experiences, frames of reference, and performance styles of ethnically diverse students to make learning encounters more relevant and effective for them, has been particularly associated with increased engagement and interest in school, as well as increased educational achievement of students who are members of underrepresented minority groups (Aronson and Laughter, 2016).

The concept of immigrant education is another idea connected to the concept of cultural diversity. Children from immigrant families are assisted as they adjust to life, academically and socially. Therefore, the teacher is one of the most significant characteristics of pre-adult entrants to a new community (Horenczyk and Tatar, 2002). Educational practices can not be successful without the support and participation of educators. Teachers are expected to alter their identities, grow as individuals, and gain knowledge from one another as part of the requirements of multicultural education (Liu and Lin, 2011).

Cultural diversity is seen as important in every country, and research has been carried out in the field of cultural diversity in educational environments in countries independently and comparatively with different countries (Forrest et al., 2017; Hordijk et al., 2019). A comparative study (Lee et al., 2020) on Russia and Taiwan, albeit under a different sub-title. A study agenda that focuses on the teacher is essential for cultural diversity. This agenda should include an inquiry into teachers’ experience with varied populations, their attitudes toward diversity, and their relevance as role models for various student populations (Horenczyk and Tatar, 2002).

Instead of using a single theoretical framework, many theoretical viewpoints were considered when analyzing the data. This was done so that the results may be interpreted in various ways. In psychology, an attitude may be defined as a collection of feelings, beliefs, and actions concerning a certain item, person, thing, or event (Cherry, 2021). Attitude consists of cognitive, emotional, and behavioral components (Breckler, 1984). To begin, the viewpoints of the educators were broken down and examined using the factors that make up attitudes. The question of whether the expressions of the instructor fell within the cognitive, emotional, or behavioral dimensions was investigated.

Garmon (2004) discovered many elements that appeared to have a crucial influence on the multicultural development of a potential teacher. Six primary elements appear to have had the most significant role in promoting the changes in teachers’ beliefs and attitudes regarding diversity. Openness, self-awareness/self-reflection, and commitment to social justice are three dispositional qualities. Openness, self-awareness/self-reflection, and commitment to social justice may be significant indicators of the likelihood that teachers may gain increased intercultural understanding and sensitivity. Other experienced variables include intercultural, educational, and support group experiences. Personal experiences with variety, as well as the chance to process these encounters appropriately, may be crucial to their development of intercultural awareness and sensitivity. In the second stage, the data were analyzed regarding dispositional and experiential factors.

In the third step, the data were examined within the context of Bryan and Atwater’s (2002). The first topic is student characteristics. Teachers’ attitudes are affected by their thoughts and opinions about the characteristics of their pupils. Teachers hold ideas about pupils from varied cultural backgrounds based on race, ethnicity, language, and socioeconomic status. The second theme is the external effects (such as parents) on learning. This area covers instructors about parental engagement, family stability, and student communities. The third element is belief in proper teacher responses to instructors from diverse backgrounds. Teachers respond to classroom situations following their ideas of appropriate behavior patterns, classroom relationships, and academic achievement.

In the fourth dimension, teachers’ views were analyzed in terms of positive, negative, and ambivalent meanings (Andreeva, 2001). According to Wiggins et al. (2007), cultivating a good attitude toward cultural diversity is essential during the training of future educators. Negative views against immigrants may vary depending on the traits or experiences of the perceivers, whether ideological, authoritarian, or experiential, such as having family members who are immigrants (Lee et al., 2020).

In the fifth phase, instructors’ perspectives were explored regarding student performance forecasts. There is a possibility that educators have stereotyped ideas, such as the perception that students attending schools with a wide range of cultural backgrounds are very disruptive and that children from ethnic minorities have poor academic performance (Castro, 2010; Glock et al., 2019).

In the last step, educators’ perspectives were scrutinized regarding equality and multiculturalism. It is strongly recommended that teachers make “equity” one of their top concerns for all of their students, regardless of the type of educational setting they are working in (Little and Bartlett, 2010). A school can be considered equitable if it raises the level of accomplishment of all its students while simultaneously closing the achievement gap between its highest-and lowest-performing pupils. Multicultural education is relevant for various lifestyles and ways of thinking, one that is respectful of otherness and one that considers the contributions of other ethnic groups to the process of individual growth (Salgur and Gursoy, 2015).

Due to the vast socioeconomic and historical disparities between the two nations, cross-cultural similarities may witness the possible cross-cultural universality of the underlying processes. In contrast, cross-cultural contrasts may provide insight into culturally particular mechanisms. On the one hand, because immigrants are frequently viewed as outsiders in many nations, we anticipate that lay views and authoritarian beliefs are associated with unfavorable sentiments against immigrants in both civilizations (Lee et al., 2020).

## Methodology

Although teachers’ attitudes toward diversity in the classroom can be measured using qualitative and quantitative methods, the literature points to the need to examine the issue with quantitative instruments (Pohan et al., 2001; Sleeter, 2001; Civitillo et al., 2018). Due to the sensitivity of topics such as attitudes toward cultural diversity in the classroom, respondents are more likely to provide socially desirable responses than in qualitative studies (Dwight and Feigelson, 2000). To examine teachers’ attitudes toward cultural diversity, we chose a qualitative methodology (Hsieh and Shannon, 2005) and used content analysis to analyze the data (Graneheim and Lundman, 2004). We used semi-structured interviews with eight questions to collect data to answer the research questions. Interview results were deductively analyzed according to the categories identified in the literature review and inductively enriched to reveal differences in these categories. Data coding and analysis were conducted using the Atlasi.ti 8 qualitative data computer program. The results were analyzed deductively according to the categories listed in the literature review and enriched inductively to clarify differences in the categories.

### Setting and participants

The participants were teachers from the Republic of Tatarstan, Russia, and Kaohsiung City, Taiwan. The Republic of Tatarstan ranks sixth in Russia regarding the number of immigrants (MIARF, 2019). The number of registered immigrants in the Republic of Tatarstan is estimated at around 126,000; 30% are labor migrants (MIARF, 2019). There are no official statistics on immigrant children in the region. In Kazan, the region’s capital, there are schools with no immigrant children and schools where immigrant children make up more than half of all students. Immigrant children learn in mainstream classes following the same curriculum as nonimmigrant children - Kaohsiung City is located in southern Taiwan. Kaohsiung City is a special municipality in southern Taiwan. It stretches from the urban center on the coast to the rural Yushan Mountains with an area of 2,952 km2 (1,140 sq. mi). Kaohsiung City has a population of about 2.77 million and is the third-largest and most populous city in southern Taiwan.

Twenty elementary school teachers, 11 from Russia and nine from Taiwan with experience working and interacting with migrant children, agreed to participate in the study. The teaching experience of the respondents ranged from 6 months to 33 years. There was a total of one male (from Russia) and 19 female teachers.

## Data collection and analysis

Each interview lasted 45 to 90 min. They were then transcribed and read by each researcher to get a general sense of the participants’ feelings and emotions and to avoid missing valuable quotes related to the research topic. Participants readily shared their experiences of working with migrant children, the specifics of their work, and their difficulties. Although we did not explicitly ask the teachers about their attitudes, these were expressed in the teachers’ remarks when they described problems and difficulties in working with migrant children. The participants from Russia were given the RP code, and those from Taiwan were given the TP code.

A deductive approach was used in coding the data. Based on the literature review, many code groups were preliminarily established for the interview analysis: (1) type of attitudes: cognitive, affective, and behavioral; (2) types of attitudes: personal and professional; (3) immigrant groups: Children, parents, different ethnic groups; (4) levels of the emotional depth of attitudes: positive, negative, ambivalent; (5) predictions of students’ performance; (6) conceptual orientation of teachers’ attitudes: equality, multiculturalism. We also inductively tried to expand the main types of characteristics that define teachers’ attitudes.

## Results

### Cognitive, emotional, and behavioral attitudes

**Figure 1:**
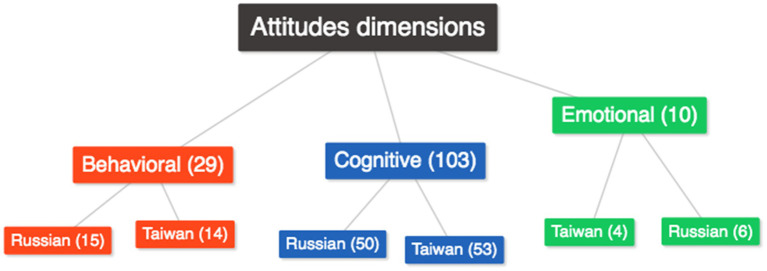
Distribution of attitudes dimensions according to countries.

First, we analyzed the interview data according to Smith et al.’s (1956) classification to determine whether participants’ cognitive, emotional, or behavioral attitudes predominated. When the participants’ attitudes are analyzed regarding their cognitive, emotional, and behavioral components, the frequency distributions across the nations are comparable for each sub-dimension (Figure 1).

The results showed that teachers’ attitudes were mainly cognitive (103 quotes), reflecting their knowledge and expectations. We further subdivided cognitive attitudes into attitudes toward:

**Table 1:** 

Students and their parents’ personal qualities	*Migrant students are very hard-working. They do not have anyone to help them at home. Their parents understand that and act accordingly. (TP)*
Immigrant students’ abilities	*I have to say, they are morally strong; nothing can break them. (RP)*
Cultural diversity	*They are all different. Everyone has their mindset. Sometimes they do not realize that they moved to another country and try to live by their own rules.(RP)* *Not every pupil must be identical. Additionally, immigrant pupils are unique. (TP)*

A much smaller group of teacher attitudes was represented by behavioral attitudes (29 citations:), which can be observed in teachers’ professional actions and guidelines for activities in culturally diverse classrooms:

Our main goal is to create a good atmosphere in the classroom so that students are not afraid of being misunderstood and do not feel ashamed. Otherwise, they might become angry and antisocial. They should not say, 'You did not understand. You are worse than others.' I think praise and compliments are necessary. (RP)

We must understand the cultures of the students and avoid offensive words. (TP)

Emotional attitudes showing teachers’ feelings and emotions toward children with cultural differences were rarely expressed (10 quotes):

We love these kids, and we feel sorry for them. It's hard for them to learn. (RP)

When I put myself in the shoes of immigrant students, I have a greater understanding of their challenges. I can assist them more. (TW)

### Dispositional and experiential factors

**Figure 2:**
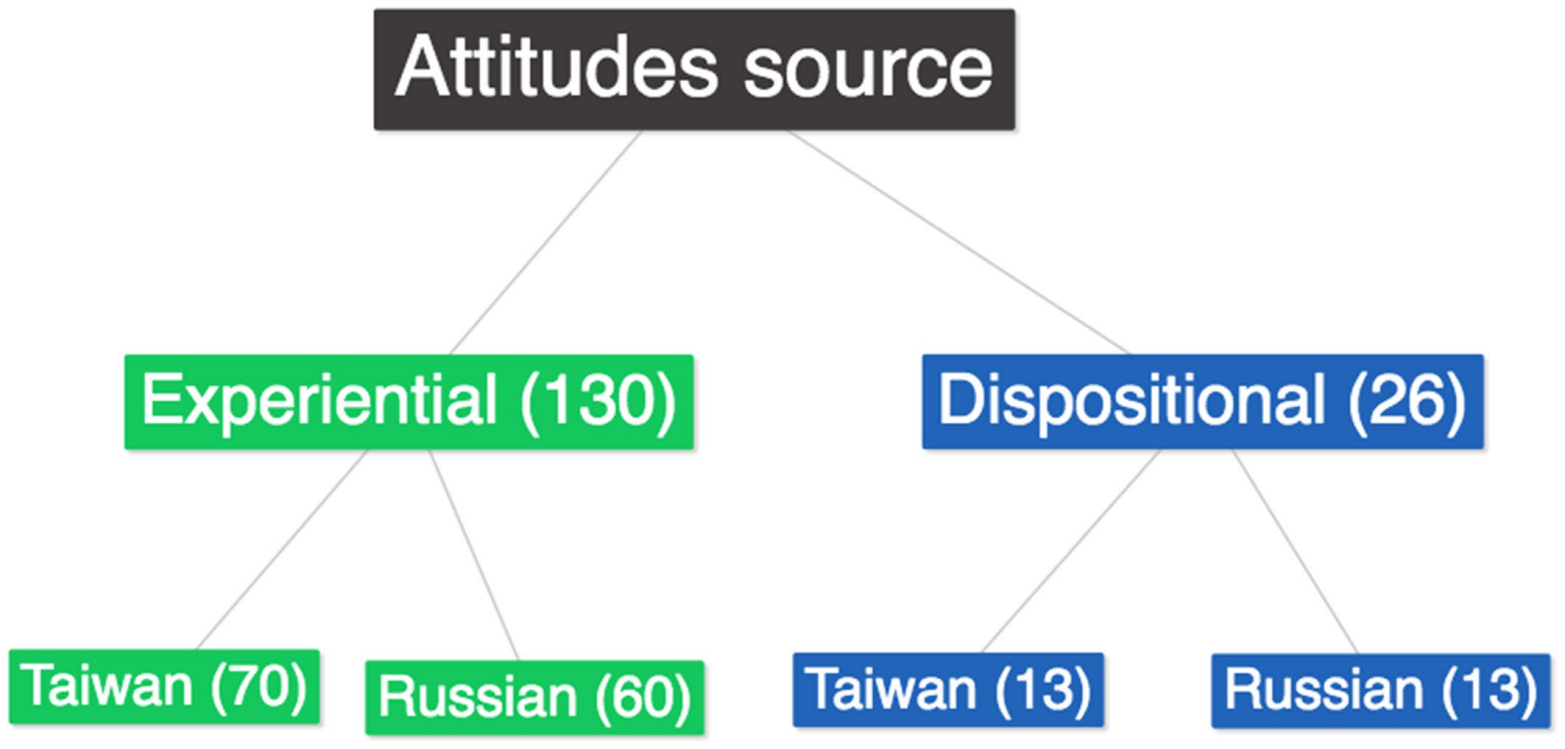
Distribution of attitudes sources according to countries.

In the second phase, we examined the data to determine whether dispositional or experiential factors (Garmon, 2004) influenced teachers’ attitudes toward diversity. The analysis revealed that teachers’ attitudes toward immigrants were primarily influenced by their professional experience, i.e., experiential factors (130 quotes). Dispositional factors were traced in 26 citations (Figure 2).

Regarding the distribution of the sources of attitudes according to the nations, there is a comparable ratio in the dispositional factors; nevertheless, when it comes to the experiential factors, Taiwanese teachers have stated it somewhat more than Russian instructors have.

Because dispositional (personal) and experiential factors can intermingle, we reviewed the data accordingly. Personal factors appeared to clash with occupational factors in 21 citations. This clash can be seen, for example, in the statements of one teacher:

The thing is that I also had difficulties with my parents. They did not want to deal with their children. Normally, I expect parents to help their children with their homework. These particular parents were migrants from Central Asia. They did not understand me, probably because they were not used to it. They left the children independently, and we always misunderstood each other. (RP)

Throughout my studies at the university, I shared the classroom with students from various nations and cultural backgrounds. We have participated in many different studies and activities with our close friends. Now, it is possible that these events were the catalyst for me to develop a more favorable outlook on the immigrant students enrolled in my class. (TP)

From this, we can tentatively deduce that the Russian teacher is not ready to accept cultural diversity (personal attitude) and believes that the requirements should be the same for all students in the class (professional attitude). However, it is understood that the experience of the Taiwanese teacher caused the teacher to exhibit a more positive attitude toward cultural diversity.

### Teachers’ attitudes toward cultural diversity

In the third data analysis phase, we categorized teachers’ attitudes toward cultural diversity according to Bryan and Atwater (2002) classification. We examined the relation between our participants’ attitudes and their beliefs about (a) student characteristics, (b) students’ parents and families, and (c) diversity. In addition, we inductively found that teachers’ attitudes toward the three groups (children, their parents, and ethnic groups in general) were inconsistent and expressed differently in respondents’ answers.

### Teachers’ attitudes related to student characteristics

According to Bryan and Atwater (2002), race, ethnicity, language, and social status are student characteristics that influence teachers’ attitudes toward cultural diversity in the classroom. We inductively extended this set of student-related characteristics based on our data:

**Table 2:** 

teacher-student relationships	*You can work with them if only they are treated equally; otherwise, they shut down. (RT)*
peer relationships in the classroom	*They join the class quietly. There are no accidents or conflicts. (TP)*
teaching and learning process	*They understand math only in terms of money. They love money. Their parents work in commerce, and that is why children understand maths better with the example of money. (RP)*
students’ knowledge of the host language	*Of course, it is difficult for teachers to interact with and teach these children. They do not know the language and do not understand what is being explained. (RB)* *Some immigrant children do not know Chinese characters and cannot communicate easily in class. (TP)*
student’s nation-specific mentality	*They respect their elders and teachers. It is their mentality. (RP)*
gender differences	*Girls do not need to be educated. Besides, the reality is that girls always perform worse than boys. (RP)*

### Teachers’ attitudes toward parents

Similarly, we have examined correlations between teachers’ attitudes and students’ parents. Bryan and Atwater (2002) added this category of “teachers’ beliefs about parental involvement, family stability, and student community” (p. 828). We expanded the category of teachers’ attitudes and identified the following groups of beliefs:

**Table 3:** 

Parents’ knowledge of the host language	*They are illiterate. I can see this in the notes that they write and send me. They cannot write a simple text message on the phone. (RP)* *Some of the parents of immigrant children do not understand Chinese characters, which prevents them from being able to review their children’s schoolwork. (TP)*
teacher-mother relationships and teacher-father relationships	*Her mother does not understand a thing in Russian. When the child comes home, there is no help. Her father is constantly working, but he often visits school after work as he is interested. (RP)* *I frequently have to spend significant time talking to the parents. I must make communication notes to keep parents informed about the manners in which their children are acting while at school. (TP)*
parents’ willingness and ability to assist their children with homework	*Parents are easy to get along with and listen to teachers’ advice. (RP)*
parents’ attitudes toward teachers	*Parents are polite. They have different customs and traditions and, thus, different attitudes to teachers. (RP)* *Parents’ attitudes toward teachers are generally positive. Parents are gentle and respectful toward teachers. (TP)*
parents’ commitment to their children’s education	*Back in the day, they used to say that if their children knew how to count money and read, it was enough. They did not need anything else. Today parents are more serious and worried.* *As a teacher, I often find that the task of educating the children of immigrant parents falls on my shoulders. This is the most significant barrier between the home and the classroom. (TP)*
Parents’ characteristics:	*Migrant parents are different from ours. They demand special attention for their children. If something goes wrong, they get offended. (TP)*
family relations	*Usually, they have nuclear families. Fathers work, and mothers stay at home with children. (RP)* *In most cases, they belong to nuclear families. In the majority of households, both the mother and the father have jobs. (TP)*
nation-specific traditions	*They have national celebrations and holidays; children do not attend school during such days. (RP)*
parents’ cultural and education level	*Most of the parents completed four grades at school. It is difficult for them to help their children. How can they possibly teach their children math or Russian? (RP)*
family’s socioeconomic status	*We know very well that our children are not very well-off financially. Most of the families have a lot of children, up to six. Many are poor… children of labor migrants. (RP)* *The majority of the families are struggling economically to a significant degree. (TP)*

**Figure 3:**
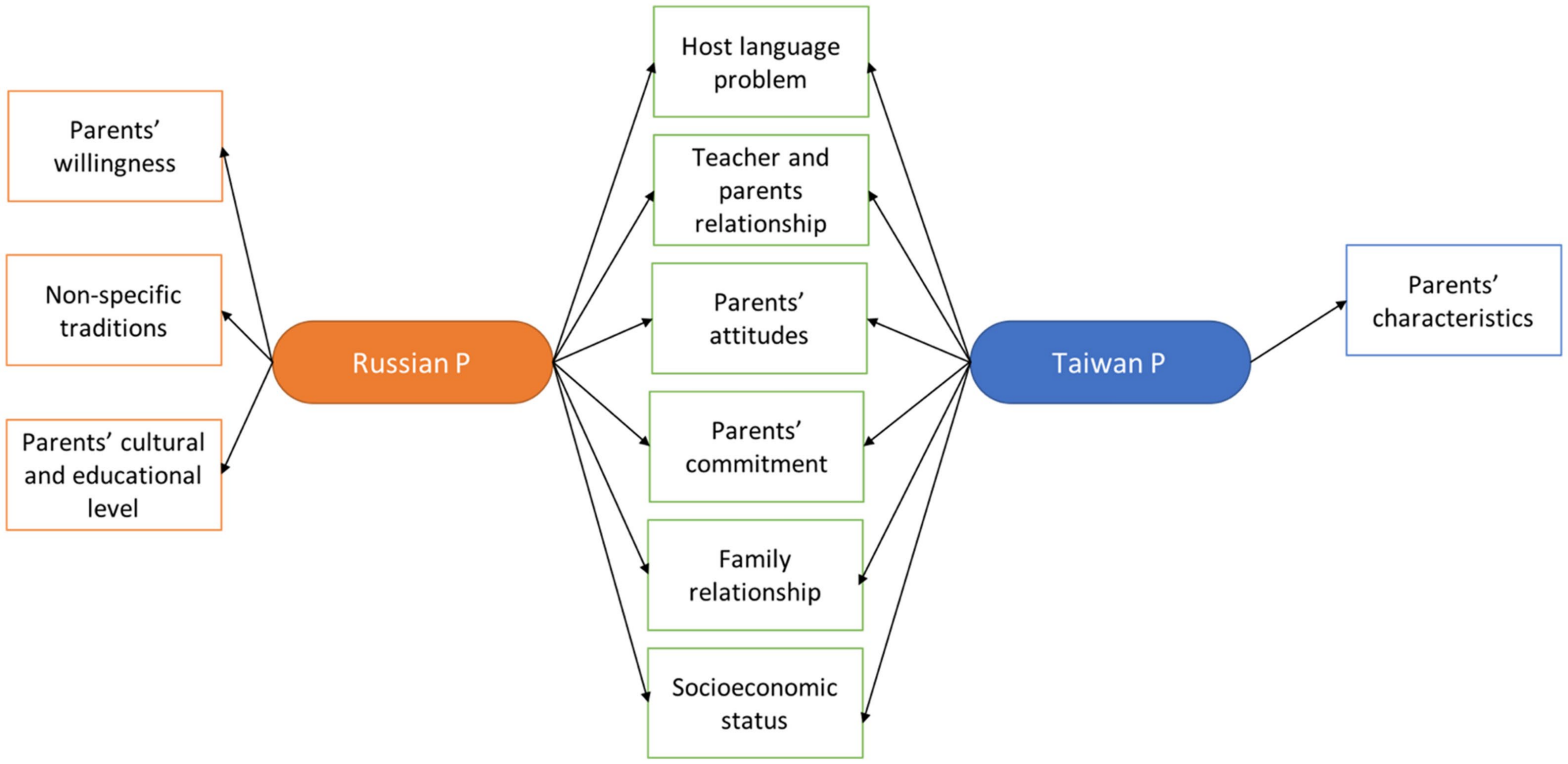
Compare teachers’ attitudes according to countries.

Common and unique themes emerge when comparing teachers’ opinions across nations (Figure 3). Examining the common themes of instructors’ thoughts toward parents reveals that the linguistic competency of parents is low in every country. Both groups of teachers have difficulties communicating with parents. In both nations, parents want teachers to assume greater responsibility for their children’s education. In addition, the teachers in Russia said that parents were willing, but their education and culture levels were low. According to Taiwanese educators, families themselves are diverse.

### Teachers’ response to diversity

Next, we examined the correlations between our participants’ attitudes and their beliefs about different ethnic groups. The in-depth analysis shows that teachers’ attitudes toward other ethnic groups are mainly based on the following factors:

**Table 4:** 

National characteristics	*Especially if we talk about Vietnamese and Koreans, they are very hard-working people who want to study. (TP)* *Tajiks are very persistent. Their culture, language, and religion, stand out among others(RP).* *I’m talking about almost all immigrants. Sometimes they distrust us. (RP)*
language skills	*Kazakhstani and Azerbaijani children are mostly good at Russian, especially if they attended kindergarten in their childhood. (RP)*
perception	*Maybe labor migrants from Asia have no pursuit of knowledge. When parents studied, they did not have a goal of finishing university. Perhaps boys in such families think they will work with their fathers at the market, and girls think they will start families and stay home with children. (RP)* *Instead of pursuing their educational objectives, most youngsters aspire to work in the business sector of their moms or honeymooners. (TP)*

Teachers have established a perception of immigrants based on their country. It is possible to say that these impressions reach the level of bias. Teachers do not value multicultural or diversity education, as seen by their attitudes.

### Positive, negative, and ambivalent attitudes

Andreeva (2001) reported that ethnic attitudes could positively or negatively affect emotional depth. Indeed, our analysis showed that teachers’ attitudes toward immigrant students were predominantly positive. At the same time, however, we could not determine the degree of emotional depth in a few quotes and labeled them as ambivalent.

Our interview data showed that teachers frequently praised the diligence and achievements of immigrant students and emphasized their high motivation to learn. Teachers emphasized that despite their difficulties with Russian, students make an effort not to fall behind the rest of the class. They seem to have more interest in learning than native students: they have a greater desire to learn than our native students. Migrant children were friendly, not cause conflicts, and participated in all school events. Teachers emphasized that migrant children are disciplined and respected by parents and teachers*: Migrant children are more disciplined and well-bred. They know the reason they attend school. They respect adults, and they have different worldviews*.

At the same time, some teachers expressed negative attitudes toward immigrant children. Our participants shared that immigrant children caused problems because of their insufficient knowledge of the Russian language and subsequent misunderstandings during classes: *Children are very hard to teach, frankly speaking.* In contrast, teachers base their assessment of student success on the students in their class. For instance, a Taiwanese educator stated, “*I do not provide additional resources for immigrant children in my class because their academic achievement is satisfactory.*.” Teachers expressed the view that learning difficulties affect students’ behavior in the classroom. They become embittered and either reserved or aggressive toward other students: *If they do not succeed in something, they get angry and start fighting, sometimes expressing their aggression.* Some teachers mentioned low motivation toward learning among girls, which accounted for women’s social passiveness in immigrant families: *That girl was very capable but lazy; she did not study. That is what they are used to getting married as soon as possible and having children.* Teachers often link immigrant students’ problems with studying and behavior to their mentality. There was an insufficient concentration of good or negative ideas to differentiate between positive and negative thoughts based on country. Both groups of instructors make both positive and negative remarks regarding immigrant students.

### Teachers’ attitudes and predictions of students’ performance

The literature review revealed that scholars who examined teachers’ attitudes toward immigrant students also investigated whether students’ immigrant and language backgrounds influenced teachers’ evaluations of student performance (Gay, 2002; Hachfeld et al., 2010). Teachers’ expectations of student academic achievement did not occur in every interview in our data set. Nevertheless, teachers expressed negative views about immigrant students’ characteristics and academic abilities and accused parents of not wanting to support their children’s learning. This group of teachers also felt that there was no need for additional tutoring for newly arrived immigrant students because the requirements should be the same for all students in the class:

Parents did not help their children at all. I asked parents to help their children write dictations in Russian twice a week and read books in Russian at home. I mean, I had the same requirements for all the students. Those students' parents did nothing. (RP)

Education standards and circumstances should be the same for all students, not less rigorous for immigrant pupils. (TP)

Low teacher expectations can also be traced to teachers’ condescending attitudes, tendency to give easier tasks, or apathy toward immigrant students: *We treated them with pity. What could you do if they cannot pull it off? You cannot kick them out; they are children, after all. (RP).*

I do not complement standard instructional materials with ones sensitive to culture. (TP)

The data showed that positive attitudes correspond to high teacher expectations and are related to teachers’ orientation toward tolerance and willingness to create conditions for children’s successful adaptation:

Children should feel comfortable. There should be a friendly environment for them so that they do not just sit and tremble. Teachers should work with them and explain everything. Children should be happy to go to school and get a good education. It is pointless to scold them. We praise them. Even small steps forward are good. (RP)

We should not disregard or appear unaware of pupils' distinctions. We should not ignore the disparities that make comparatively disadvantaged pupils uneasy. (TP)

Teachers emphasized that many immigrant children are motivated to learn. Motivation comes from families, and although parents might have a low level of education, they still want their children to succeed so that they can live and work in Russia: *Parents understand that their children have more opportunities to get an education in our school and then, live and work here (RP)*. According to the interview data, parents respect teachers and listen to their advice, especially concerning fathers who are reported to be more interested in their children’s education. That said, teachers also admitted that despite their will, parents could not assist their children with studying for two main reasons – parents’ very low level of education coupled with insufficient knowledge of the Russian language and financial instability.

Teachers believe many difficulties in educating immigrant children are caused by parents often moving in search of a better placement. Children are forced to drop their studies in the middle of the school year and start anew: *The parents’ business was not successful, and they left in late December. They took children and left when you thought that they had just started getting into the learning process. I wasted my energy on them (RP).* Some immigrant children do not attend school at all. Girls stay at home and help their mothers in housekeeping, and boys work with their fathers at markets:

One of my students is brought to school by his sister. She is two years older than him, she is 12 years, but she stays at home even if she is of school age. Her parents are temporarily here [in the city] and did not enroll her anywhere. How could this be happening today?! (RP)

Some students drop out of school because they must work at a young age. (TP)

### Conceptual orientation of teachers’ attitudes: Equality and multiculturalism

As discussed in the literature review, Civitillo et al. (2017) identified two approaches to cultural diversity in schools: Promoting Equity and Promoting Cultural Pluralism. During data analysis, we found that specific teacher quotes reflected the conceptual orientation of teachers’ attitudes toward cultural diversity in the classroom. In this way, cultural pluralism (multiculturalism) was manifested in the interviews of half of the participating teachers. Orientation to multiculturalism means preserving the unique cultural identities of all ethnic groups. In the educational context, this strategy views students’ cultural identities as a positive and important element in teaching and learning. Multiculturally oriented teachers build the teaching process around the cultural background of immigrant students:

Yesterday we held the school event. Everyone who wanted to perform. Those immigrant children, who do not speak the language [Russian], drew pictures. Some of them recited poems and sang songs in their native languages. (RP)

Teachers' stance is very important. The teacher should teach children to respect every nationality. (RP)

Multicultural education teaches students to comprehend cultural differences, respect others and their differences, bridge the gap between them, acquire knowledge of many cultures, and cultivate cultural awareness. (TP)

The analysis of the interviews showed that the teachers are mainly multicultural-oriented and believe that immigrant children need a special approach that considers the students’ linguistic and psychological differences when adapting to a new learning environment. Teachers stressed the importance of involving students in various events where they can showcase their talents and introduce their different cultures to their peers. They agree that the history and culture of immigrant children’s home countries should be respected. This way, all students can learn to respect their own culture and those of other nations in a multicultural classroom environment.

The conceptual orientation toward equality (inclusion) was less evident than cultural pluralism, but some teachers expressed their belief that:

We teach them like all the others. No differences between ours and theirs. In any case, these children should not differ from others. We should see that they do not form separate groups in school that can grow into gangs later. That is terrorism and extremism prevention. (RP)

We should not disregard or appear unaware of pupils' distinctions. We should not ignore the disparities that make comparatively disadvantaged pupils uneasy. (TP)

The focus on equality emphasizes the importance of treating all people equally, regardless of their background. In the educational context, this strategy suggests minimizing cultural differences to focus on finding commonalities among students from different cultural backgrounds (immigrant and native) and promoting inclusion for all. Equity-minded teachers have mixed feelings about diversity. They claim that their classes have no cultural differences and that the only ‘nationality’ is ‘the student.’ Teachers believe that immigrant children are no different from native students and that the requirements should be the same for all students.

Moreover, equality-oriented teachers say it is not worthwhile to distinguish between immigrant and native students because the former might feel inferior otherwise. At the same time, some respondents admit that they are forced to work with immigrant students because it is difficult to expel them from school. Teachers believe that immigrants should understand that when they come to another country, they should live by the rules of that country and not the other way around. Some teachers emphasize that schools should create conditions, so students do not form ethnic groups to prevent possible cases of extremism.

## Discussion and conclusion

According to the three-component structure of attitudes (Smith et al., 1956), the attitudes of the participating teachers seem to be manifested mainly at the cognitive level. However, some interview comments illustrate the behavioral nature of attitudes. In making this statement, we do not claim to be impartial, as the behavioral nature of teachers’ attitudes toward cultural diversity is still debated in social psychology (Gay, 2013). Moreover, according to Rokeach’s (Rokeach, 1968) classification, cognitive attitudes are mostly descriptive and evaluative, which was also the case with our participants’ comments. This information indicates that teachers’ attitudes toward cultural diversity in the classroom are mostly cognitive in our sample. When the country-specific frequencies of the attitude components were analyzed, it was shown that they were comparable.

Our research findings are also consistent with Garmon’s (Garmon, 2004) conclusions that teachers’ attitudes toward cultural diversity are based on both dispositional (personal) and experiential (professional) factors, which may be intermingled. Although Pohan (1996) suggests that personal attitudes have a greater influence on teachers’ acceptance of diversity in school, we were unable to determine in our qualitative study how and to what extent personal and professional attitudes influence teachers’ attitudes toward cultural diversity. Regarding the distribution of the origins of attitudes by nation, there is a similar proportion of dispositional elements; however, when it comes to experience factors, Taiwanese teachers have mentioned it more frequently than Russian instructors. According to Aragona-Young and Sawyer (2018), teachers’ familiarity with multicultural learning is crucial. It is simpler to prepare a multicultural and diverse education for the classroom when teachers have personal experience. Also, teachers who worked in schools with greater diversity had less implicitly negative sentiments toward the pupils of minority groups (Glock et al., 2019).

Another important finding of our study is that we inductively extended and systematized Bryan and Atwater’s (2002) classification of teachers’ attitudes toward cultural diversity.

The following student-related characteristics should be considered: teacher-student relationships, peer relationships in the classroom, the teaching and learning process, students’ knowledge of the host country’s language, students’ country-specific mentality, and gender differences. The issues that arise with pupils’ linguistic competence are the primary topic of conversation for teachers who work with immigrant kids. For instance, educators participated in the research by Sinkkonen and Kyttälä (2014), reporting that pupils lacked sufficient Finnish language knowledge. According to the results of Hachfeld et al. (2010)‘s study, high language skills rather than ethnic background were more effective in overestimating students’ performances.

Then, teachers’ attitudes toward parents and immigrant families can be considered based on parents’ knowledge of the host language, teacher-mother and teacher-father relationships, parents’ willingness and ability to help their children with homework, parents’ attitudes toward teachers, parents’ commitment to their children’s education, parents’ characteristics, family relationships, country-specific traditions, parents’ cultural and educational levels, and family socioeconomic status. When comparing instructors’ perspectives across nations, universal and specific elements emerge. Examining the common themes of teachers’ attitudes toward parents indicates that the language competence of parents in every country is low. Both types of educators struggle to communicate with parents. In the study by Yang et al. (2014), Taiwanese teachers reported having issues with the families of newly enrolled Taiwanese pupils. The findings of the study (Khairutdinova et al., 2019) that was carried out in Russia indicate that the social difference between teachers and immigrants from countries in the Caucasus and Central Asia is the highest. Educators in their home nations may be more intolerant of certain immigrants. For instance, it has been said that there is a certain amount of intolerance and discrimination toward Aboriginal and LBOTE Australians among the teaching profession and the general population (Forrest et al., 2017). In both nations, parents want teachers to bear more responsibility for their children’s education. In addition, instructors in Russia said that parents were willing but that their educational and cultural backgrounds were lacking. Educators in Taiwan assert that families themselves are different. These findings parallel the study (Yang et al., 2014). According to Lee et al. (2020), it appears that the attitude of Taiwanese people toward immigration is solely motivated by ideology. On the other hand, Russians’ attitudes toward immigrants appear to be partially based on personal experiences. This is because the associations between a person’s background, such as their age and whether or not they have relatives who are migrants, significantly predict how they feel about immigrants.

Teachers’ attitudes toward ethnic groups and cultural diversity were noted in many comments. This category of attitudes should be analyzed through the prism of teachers’ perceptions of a child or adult immigrant and national characteristics, language proficiency, and perception. The educators have formed an opinion about immigrants depending on the nation in which they were born. One may argue that these perceptions are so strong that they constitute prejudice. As may be seen from their opinions, educators do not place a high emphasis on multicultural or diversity education.

Teachers’ attitudes toward parents and different ethnic groups seemed ambivalent and negative. For this reason, we should also mention the results of our previous research, in which we determined the social distance of teachers toward different ethnic groups at the social level. A certain trend shows that teachers have more positive attitudes toward cultural diversity in society than in their classes (Horenczyk and Tatar, 2002; Khairutdinova et al., 2019). Many educators have unfavorable attitudes about students of ethnic minorities (Glock et al., 2019). According to McCorkle (2020), the fact of the matter is that it is challenging to alter the perspectives of educators on a topic as touchy and controversial as immigration.

In examining the emotional depth of teachers’ attitudes and their relationship to predicting students’ academic achievement, we found that positive attitudes lead to high expectations and, on the contrary, negative attitudes lead to low expectations. Gay (2002) and Hachfeld et al. (2010) reached similar conclusions in their studies. According to the results of the study (Glock et al., 2019) conducted in Germany, teachers attribute the low academic achievement of Turkish students to lower ability and effort.

Although our interviews were not designed to explore the conceptual orientation of cultural diversity attitudes, it was evident in some teachers’ comments, which revealed that teachers were primarily multicultural in orientation. We can conclude that greater levels of intercultural contact lead to more tolerant approaches that teachers draw upon in their teaching. Fine-Davis and Faas (2014) reported similar findings when they examined teachers’ attitudes in some European countries. However, in some interviews, some comments reflected a conceptual focus on equity. Apfelbaum et al. (2010) suggested that the equity-oriented approach and equity-minded teachers can promote broader student involvement. At the same time, we believe that this conceptual approach is difficult to interpret unambiguously and should be more closely examined and systematized, as attempted by Milner (2010), who outlined five ‘conceptual repertoires of teachers concerning diversity (p.118), namely colorblindness, cultural conflict, meritocracy, deficit perceptions, and expectations. However, more extensive research is needed.

The attitudes of Russian and Taiwanese instructors toward immigrant pupils and their families were investigated. Interviews were employed to acquire data for the study. Examining the country-specific frequencies of the attitude components revealed that they were comparable according to the study’s findings. In both nations, the cognitive component is emphasized. We might speculate that the Russian instructor cannot recognize cultural variation (personal attitude) and feels that all students should be held to the same standards (professional attitude). However, it is known that the Taiwanese teacher’s prior experiences have led to a more welcoming attitude toward cultural diversity. For instructors working with immigrant children, linguistically-related issues are a key topic of discussion. In their nations, educators are less accepting of some immigrants. Educators have acquired opinions on immigrants based on their country of birth. One may argue that these impressions are powerful enough to foster bias. Numerous educators hold unfavorable views about kids from ethnic minorities. Positive views toward student accomplishment result in high expectations, while negative attitudes result in low expectations. Higher levels of intercultural contact result in instructors employing more tolerant methods of instruction.

## Limitations and implications

Research on teachers’ attitudes toward immigrant children is critical because it determines teachers’ classroom behavior. Further research should focus on developing a measure that encompasses various aspects of teachers’ attitudes. Conceptual orientation related to cultural diversity also needs a more detailed analysis.

One limitation of our study is that it was conducted in only one region of the Russian Federation, namely the Republic of Tatarstan, which is considered one of the most multicultural regions in the country. The results may be different in other Russian regions. Moreover, our respondents were elementary school teachers who deal with children all the time, as opposed to secondary school subject teachers who deal with students less frequently. For this reason, an examination of elementary and secondary teachers’ experiences working with immigrant children could yield different results.

Research findings have shown that teachers must have enhanced skills and competencies to work effectively in a new, culturally diverse environment to facilitate more inclusive instruction. Future research will identify the categories of teachers’ attitudes reflected in instructional practice in a multicultural classroom and determine how teachers’ attitudes influence their pedagogical practice in a multicultural classroom.

## Data availability statement

The raw data supporting the conclusions of this article will be made available by the authors, without undue reservation.

## Ethics statement

The studies involving human participants were reviewed and approved by the ethical committee of I-Shou University. The ethics committee waived the requirement of written informed consent for participation.

## Author contributions

All authors listed have made a substantial, direct, and intellectual contribution to the work and approved it for publication.

## Funding

This paper has been supported by the Kazan Federal University Strategic Academic Leadership Program (Priority-2030).

## Conflict of interest

The authors declare that the research was conducted in the absence of any commercial or financial relationships that could be construed as a potential conflict of interest.

## Publisher’s note

All claims expressed in this article are solely those of the authors and do not necessarily represent those of their affiliated organizations, or those of the publisher, the editors and the reviewers. Any product that may be evaluated in this article, or claim that may be made by its manufacturer, is not guaranteed or endorsed by the publisher.
